# First experimental evaluation of the alpha efficiency in coarse-grained quartz for ESR dating purposes: implications for dose rate evaluation

**DOI:** 10.1038/s41598-019-54688-9

**Published:** 2019-12-24

**Authors:** Melanie Bartz, Lee J. Arnold, Nigel A. Spooner, Martina Demuro, Isidoro Campaña, Gilles Rixhon, Helmut Brückner, Mathieu Duval

**Affiliations:** 1 Institute of Geography, University of Cologne, Albertus-Magnus-Platz, 50923 Cologne, Germany; 2 School of Physical Sciences, Environment Institute, and Institute for Photonics and Advanced Sensing (IPAS), University of Adelaide, North Terrace Campus, Adelaide, SA 5005 Australia; 3 Defence Science and Technology Group, Third Avenue, Edinburgh, SA 5111 Australia; 4 Centro Nacional de Investigación sobre la Evolución Humana (CENIEH), Paseo Sierra de Atapuerca, 3, 09002 Burgos, Spain; 5 ENGEES/Laboratoire Image Ville Environnement (LIVE), UMR 7362 - CNRS (University of Strasbourg), Quai Koch 1, 67000 Strasbourg, France; 6 Australian Research Centre for Human Evolution (ARCHE), Environmental Futures Research Institute (EFRI), Griffith University, 170 Kessels Road, Nathan, QLD 4111 Australia

**Keywords:** Environmental sciences, Scientific data

## Abstract

We present the first experimental evaluation of the alpha efficiency value for electron spin resonance (ESR) dating of coarse quartz grains, which is used for the evaluation of the internal and external alpha dose rate components. Based on our results, we recommend the use of an a-value of 0.07 ± 0.01 (1σ) for both the Al and Ti centres. Although we acknowledge that quartz ESR alpha efficiency may be sample dependent, and could also be impacted by other sources of uncertainty, this potential variability is presently impossible to evaluate given the absence of other experimental a-values available in the ESR dating literature. Measured radioactivity of quartz grains from the Moulouya catchment (NE Morocco) provides an internal dose rate in the range of 50–70 µGy/a when using an a-value of 0.07. The use of this empirically derived a-value for the evaluation of the internal and external alpha dose rate has a limited overall impact on the final ESR age results: they change by <2% and <3%, respectively, in comparison with those obtained with an assumed a-value. However, the large variability observed among the broader sample dataset for quartz internal radioactivity and hydrofluoric acid (HF) etching rates underscores the potential importance of undertaking experimental evaluations of alpha dose rate parameters for each dated sample.

## Introduction

For both ESR and luminescence dating of quartz grains, the relative efficiency of alpha particles in producing a signal compared to beta particles or gamma rays is a key parameter for the evaluation of long-term environmental dose rates^1^. Generally, the alpha efficiency parameter affects the calculation of two main dose rate components: the internal and external alpha dose rate. The former is frequently assumed in quartz dating studies (e.g.^2–4^), or even neglected given the low radioelement content usually measured in quartz grains (e.g.^5,6^). In comparison, in most coarse-grained (either >63 μm or >90 μm) quartz dating studies the external alpha dose rate is considered virtually null when the outer layer of the grains has been removed by hydrofluoric acid (HF) etching. Consequently, the contribution of the internal and external alpha components to the total dose rate is usually overall assumed to be minimal (<10%, e.g.^7^), as it often falls within the 2σ-uncertainty range of the dose rate estimate. In contrast, for fine-grained (typically 4–11 μm) luminescence dating studies, it is impractical to remove the alpha irradiated rinds of individual grains using HF etching. Hence, the external alpha dose component and the alpha efficiency parameter are routinely considered for dose rate evaluation^1^. While a significant body of research has focused on determining accurate estimates of quartz alpha efficiency for optically stimulated luminescence (OSL) and thermoluminescence (TL) dating purposes (e.g.^8–15^), this subject has received little attention in the ESR dating community in comparison with other sources of systematic uncertainty affecting beta and gamma dose rates. Indeed, there are presently no empirical estimates of quartz alpha efficiencies reported in the literature for ESR dating. Since the first ESR dating application to optically bleached quartz grains^16^, the alpha efficiency value has been systematically assumed (e.g.^4,17–20^). Moreover, there have not been any detailed assessments of the potential age offsets that might arise from adopting assumed (or neglected) alpha dose rate components in ESR dating studies.

In order to address this knowledge gap, we have performed a series of comparisons between alpha and gamma irradiations for two coarse-grained quartz ESR samples from the Moulouya catchment in NE Morocco^20^. The ESR measurement of these samples has enabled us to derive the first experimental estimate of the alpha efficiency for ESR dating of quartz. Here we present the results of these experiments and discuss their implications for the evaluation of ESR internal and external dose rates. We also use our empirical assessments to assess the validity of some of the standard assumptions and considerations made around internal and external alpha dose rate components in routine ESR dating studies.

## Alpha Efficiency In Luminescence and ESR Dating of Quartz

Different systems have been developed to evaluate the effectiveness of the alpha particle contribution (see an overview in^1^). Among others, the k*-*^8^ and the a*-*^9^ values are most frequently used to assess the alpha efficiency for quartz dating applications. The k-value is defined as follows^1^:

$${k}_{3.7}=\frac{ESR\,\mathrm{int}\,ensity\,per\,Gy\,for\,3.7\,MeV\,alpha\,particle\,irradiation}{ESR\,\mathrm{int}\,ensity\,per\,Gy\,for\,beta\,particle\,or\,gamma\,ray\,irradiation\,}$$

where the a-value is equivalent to the k-value when using an alpha source delivering 3.7 MeV alpha particles to a thin layer of quartz^1,21^. However, the alpha particles received during the past in nature have likely less energy than 3.7 MeV and therefore k_eff_ has been introduced^8^, where a = k_eff_ = k_3.7_ * 0.83 (for a sample with equal Th and U activities)^22^.

The first experimental evaluation of the a-value in optical dating is reported by^12^. After this initial work, the alpha efficiency in fine quartz grains (4–11 µm) has been thoroughly evaluated in luminescence dating using the a-value system (eg.^9^). Reported values usually range between 0.03 and 0.06. For example, measured alpha efficiency values of between ~0.032 and ~0.043 were obtained with the multiple-aliquot additive-dose (MAAD) method for fine-grained fluvial samples^11^. Using the single-aliquot regenerative (SAR)-dose technique^23^, an a-value of 0.04 was determined from marine sediments^13^. Similarly, an average a-value of 0.03 was obtained for silt-sized quartz samples from different depositional contexts^14^, showing thus that the alpha efficiency value may be independent of sample origin. A similar conclusion was reached for Chinese loess samples, with an inferred average alpha efficiency value of 0.035^15^. In contrast, other works stated that the quartz alpha efficiency value in TL dating appears to be sample dependent and should thus be measured in each sample (eg.^24^). Moreover, differences in the behavior of OSL and TL growth curve and thus variations in the alpha sensitivity, have been observed^25^, with the former showing lower values compared to those of the TL signal. They may potentially be due to non-identical traps and luminescence centres. In comparison, the alpha efficiency of coarse-grained (>40 µm) quartz grains has been poorly investigated in OSL dating studies and only few works exist where the a-value has been experimentally derived. For example, measured average a-values of 0.04 have been published for the 90–125 μm grain size fraction^10,26^, while assumed values have also been reported (e.g., 0.10 ± 0.02 in^27^).

In ESR dating, the majority of studies deal with coarse quartz grains >50 µm, and mostly between 100 and 200 µm. Larger grains are likely characterized by stronger ESR signals of both Al and Ti centres, while fine grains do not systematically show Ti signals^28^. They also show higher bleaching efficiency^29^. Consequently, coarse grains >100 µm are usually considered as the most appropriate for ESR measurements (eg.^30^). A k-value of 0.2 ± 0.1 was first assumed for an ESR dating study of aeolian and littoral quartz grain samples (40–160 µm) based on the MAAD method^16^. This value has subsequently been adopted in several coarse-grained (60–200 µm) ESR dating studies (eg^17,18,20^). Similarly, a k-value of 0.15 ± 0.1 has been assumed for fluvial and littoral sediments from France and Spain^4,19^. Although not specifically stated in^16^ and^19^, we interpret both k-values proposed in the two studies as being equivalent to a-values of 0.17 ± 0.08 and 0.12 ± 0.08, respectively. One may note the large relative uncertainty (around 50%) considered by the authors, which is reasonably assumed to cover the true alpha efficiency value. In another review paper, the value is assumed to be higher than that derived for TL studies^31^, (p.94: “The alpha-efficiency is routinely determined by TL investigations at around 0.1 (see^8^). However, the value for ESR dating might be greater.”), but this hypothesis is actually not based on any experimental evidence.

In summary, all the ESR dating studies published so far are based on assumed alpha efficiency values. Consequently, the true alpha efficiency of coarse-grained quartz has never been experimentally determined and is simply unknown for this dating technique.

## Material and Method

### Sample preparation and irradiation procedure

This study focuses on two quartz samples (C-E3886 and C-E3891, initial grain size 100–200 µm) from fluvial terrace sediments deposited by the Moulouya River during the Early Pleistocene in its lowermost sedimentary basin (i.e., the Triffa Plain in NE Morocco)^20^. These samples have been specifically selected because (i) they have been previously ESR dated following the multiple centre (MC) approach and using the MAAD method^20^ (full details about chemical pretreatment, mineral separation and HF etching can be found therein), and (ii) successful comparative dating studies have been undertaken on similar, contemporaneous alluvial sections in this sedimentary basin^32^.

For each quartz sample, a set of two alpha- and two gamma-irradiated aliquots was prepared. Gamma irradiations were carried out at the Centro Nacional de Investigación sobre la Evolución Humana (CENIEH, Burgos, Spain) using a calibrated Gammacell-1000 ^137^Cs gamma source (dose rate = 6.80 ± 0.15 Gy/min). Two aliquots of each sample (with an initial, pre-HF etching, grain size of 100–200 µm) were gamma irradiated to 200 and 395 Gy as part of the previous dating study^20^. Alpha irradiations were carried out at the Prescott Environmental Luminescence Laboratory, University of Adelaide, Australia, using a calibrated Littlemore 6-source ^241^Am alpha source (emitting 3.7 MeV alpha particles) that provided mean track lengths in the dosimeter of 0.704 μm^−2^ min^−1^. This source delivered a dose rate of 8.92 ± 0.27 Gy min^−1^ to each sample at the time of irradiation, taking into consideration the radioactive decay of the ^241^Am source (t_1/2_ = 432.2 yr) since its original calibration (August 1982). Due to the low penetration range of alpha particles^1^, the two aliquots of each sample were manually ground to <11 µm with an agate mortar and pestle. A centrifuge was then used in order to remove grains <4 µm, resulting in a final grain size of 4–11 µm for the alpha-irradiated aliquots. A pipette was used to achieve the deposition of a monolayer (1.0 ± 0.1 mg) on aluminium discs (9.8 mm in a diameter). Alpha irradiation was conducted under a vacuum, with six aluminium discs irradiated simultaneously (the dose rate to each disc can be considered equal owing to the irradiator design and disc configuration). In total, ~25 mg of 4–11 µm quartz was alpha irradiated per aliquot, with two alpha doses of 2230 and 4460 Gy administered to the two separate aliquots, i.e. an alpha dose approximately 10 times higher than their gamma-irradiated equivalent.

### ESR measurements

**Figure 1:**
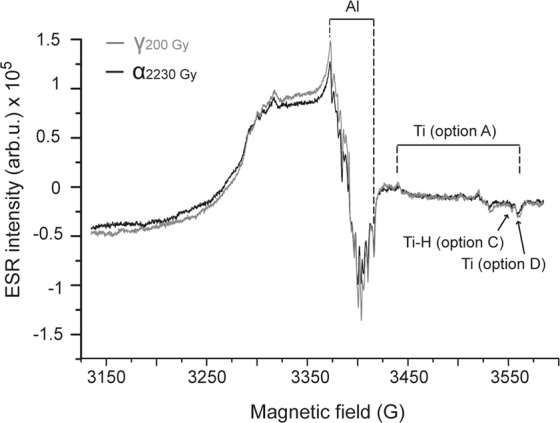
ESR spectra of gamma- (~200 Gy) and alpha- (~2230 Gy) irradiated aliquots of sample C-E3891.

In order to replicate the same experimental conditions used for the alpha-irradiated aliquots, the coarse-grained gamma-irradiated aliquots were similarly ground to 4–11 µm prior to ESR measurements. An EMXmicro 6/1 Bruker X-band ESR spectrometer coupled to a standard rectangular ER4102ST cavity and a ER4141VT Digital Temperature control system based on liquid nitrogen cooling was used for ESR measurements at CENIEH (see^33^ for further details about the experimental setup and its stability). The intensity of the aluminium (Al) and titanium (Ti) centres were measured in one single spectrum using the following parameters: 5 mW microwave power, 2048 points resolution, 100 kHz modulation frequency, 1 G modulation amplitude, 450 G sweep width, 40 ms conversion time and 10 ms time constant. Given the limited amount of material available (between 21 and 27 mg, depending on the aliquot considered), the number of scans was set to between 25 and 35, resulting in up to 50 min-long measurements per aliquot. Examples of ESR spectra are displayed in Fig. 1. The gamma- and alpha-irradiated equivalent aliquots of each sample were successively measured in order to minimize the uncertainty due to short-term drift of the ESR spectrometer. This procedure was repeated several times over different days (up to five) to evaluate the variability of the measurements. The Al ESR intensity was measured between the top of the first peak (g = 2.018) and the bottom of the 16^th^ peak (g = 1.993)^34^. The Ti ESR intensity was extracted from three different options^35^: (i) peak-to-peak amplitude measurement between g = 1.979 and the bottom of the peak at around g = 1.913 (option A, Ti centre); (ii) peak-to-baseline amplitude measurement around g = 1.913 (option D, Ti centre) and (iii) peak-to-baseline amplitude measurement around g = 1.915 (option C, Ti-H centre) (Fig. 1). Note that no baseline correction was performed on the ESR spectra before extracting the ESR intensity. The bias induced by the drift of the spectrometer over different days of measurements (due to slightly different tuning conditions) was removed by correcting the overall apparent shift of the ESR intensities observed between the successive repeated measurements. A mean ESR intensity and associated standard deviation was derived from the repeated measurements. These values were then normalized to a 1 Gy dose as per the definition of the a-value, and the ratio of the normalized alpha-irradiated ESR intensity to the normalized gamma-irradiated ESR intensity was calculated. The a-value 1-σ error has been obtained from the combination of the standard deviation of the ESR measurements and the error on the gamma and alpha source dose rates (2.2% and 3.4%, respectively).

## Results

The gamma-irradiated aliquots exhibit systematically higher ESR intensities than their alpha-irradiated equivalents for both the Al and Ti centres, although the alpha irradiation doses were chosen to be approximately ten times higher (~2230 and ~4460 Gy) than the gamma dose values (~200 and ~395 Gy) (Table S1). This is a good illustration of the lower efficiency of alpha irradiation in producing ESR signals when compared to gamma rays. Similar observations have been reported for OSL studies (e.g.^14^), when comparing dose-normalized OSL signals obtained using alpha and beta irradiation.

Empirical a-values were obtained for samples C-E3886 and C-E3891 from the ratio of the dose-normalized ESR intensities of the alpha-irradiated aliquot to that of the gamma-irradiated aliquot. The numerical values associated with the two dose steps of each sample are given in Table 1, along with a summary of the a-value results. The Al signal a-values vary within a narrow range from 0.072 to 0.076 and have a mean a-value of 0.075 ± 0.002. The limited variability (2.4%) most likely reflects the high signal-to-noise (S/N) ratio of the Al signal, which ensures good measurement repeatability. The different options of the Ti centres, which display much weaker ESR intensities compared to that of the Al centre^35^, show higher variability in their calculated a-values (between 5.4% and 15%). The mean a-values of the various Ti centre options are nevertheless within error of each other, yielding values of 0.068 ± 0.004, 0.074 ± 0.011 and 0.074 ± 0.009 for options A, C (Ti-H) and D, respectively. As the mean a-value results obtained for the Al and Ti centres are statistically indistinguishable, it is appropriate to calculate a mean value of 0.073 ± 0.003 for the combined dataset as a reliable estimate of the true alpha efficiency for the ESR Al and Ti signals measured in these quartz samples. Given the associated uncertainty on the individual results, we recommend the use of a rounded a-value of 0.07 ± 0.01 (1σ) for future ESR dating applications. This value is roughly half of the alpha efficiency value assumed (0.15–0.2, corresponding to 0.12–0.17 in the a-value system) in previous studies (e.g.^16,19^). This value, though in the same order of magnitude, is higher than the alpha efficiency a-value measured in fine-grained quartz OSL studies (around 0.04; e.g.^13–15^.

**Table 1:** a-values obtained for the measured Al and Ti centres of the two studied samples C-E3886 and C-E3891.

Sample ID	α-irradiation dose (Gy)	γ-irradiation dose (Gy)	a-value			
			Al centre	Ti centre (Option A)	Ti-H centre (Option C)	Ti centre (Option D)
C-E3886	2230 ± 66	200 ± 5	0.076 ± 0.004	0.068 ± 0.004	0.068 ± 0.006	0.071 ± 0.005
C-E3886	4460 ± 132	395 ± 9	0.072 ± 0.006	0.071 ± 0.005	0.084 ± 0.004	0.081 ± 0.003
C-E3891	2230 ± 66	200 ± 5	0.076 ± 0.003	0.071 ± 0.003	0.082 ± 0.008	0.081 ± 0.003
C-E3891	4460 ± 132	395 ± 9	0.075 ± 0.003	0.063 ± 0.002	0.061 ± 0.003	0.063 ± 0.003
Average a-value and associated error (1 s.d.)			0.075 ± 0.002 (2.4%)	0.068 ± 0.004 (5.4%)	0.074 ± 0.011 (15.0%)	0.074 ± 0.009 (11.8%)

Finally, it is worth mentioning that applying a baseline correction to the ESR spectra has virtually no impact on the mean alpha efficiency value: it slightly decreases by 0.4% but remains statistically undistinguishable with the original value of 0.073 ± 0.003 obtained without baseline correction.

## The Role of Alpha Efficiency in Dose Rate Evaluation

Alpha efficiency plays a significant role in the evaluation of both the internal and the external alpha dose rate components^1^. In ESR and luminescence dating of coarse quartz grains, an important consideration is whether these two components can reasonably be: (i) neglected, (ii) assumed in the dose rate evaluation, or (iii) should be systematically derived from experimentally collected data for each dated sample. To better evaluate the impact of our newly obtained alpha efficiency value on ESR dose rate evaluations, we have performed a series of additional analyses [Inductively Coupled Plasma-Mass Spectrometry (ICP-MS), Static Image Analyses (SIA) and Scanning Electron Microscope (SEM); Supplementary Information] on four quartz samples from the Moulouya area (samples C-E3824, C-E3886, C-E3888 and C-E3891)^20^. These newly-obtained experimental data provide some key insights about grain size and shape, internal radioactivity of quartz grains, or HF etching at both multi- and single-grain levels, which are all essential parameters in the internal and/or external alpha dose rate evaluation.

### Influence of grain morphology and HF etching on dose rate considerations

Circularity, aspect ratio and convexity values vary within narrow ranges for the four natural samples (0.827–0.843, see Table S2), suggesting that they overall show similar grain shapes. Mean circularity and aspect ratios are of 0.834 and 0.764, respectively, showing that the grains cannot be approximated to circles. Convexity values are instead close to 1 (0.977 on average), indicating the limited edge roughness of the grains. Despite similar grain shapes for the four samples, the mean grain size measured in the 100–200 µm fraction shows much higher variability: two samples have close mean CE diameters of around 185 µm (C-3886 and C-3891), while samples C-E3824 and C-E3891 display significantly higher and lower values, respectively (Table S2).

These observations are consistent with those from a previous SIA study, which involved consideration of a larger set of samples from different origins^36^. The 2D images show that the quartz grains from the Moulouya samples are, in general, not perfect spheres (i.e. the 3D analogue of a circle), but rather should be approximated as smooth and slightly elongated ellipsoids (i.e. the 3D analogue of an ellipse), with a width that is on average about 25% smaller than the length. These shape characteristics may potentially impact both the internal and external dose rate components (see next sub-sections).

As might be expected, HF etching induces significant modifications in the grain size and shape of the four samples. Overall, etching adds more variability in each of the grain shape parameters, as indicated by the increase of the coefficient of variation (from 0.2% to 0.4% for convexity; from 0.8% to 1.3% for circularity; from 0.6 to 1.5% for the aspect ratio; Table S2). This may be interpreted as evidence of uneven HF etching among the grains. Additionally, mean convexity drops from 0.977 to 0.961, indicating a reduced edge smoothness for the grains. This suggests that HF does not homogeneously etch the surface of the grains. Additionally, the circularity significantly decreases after HF etching (from 0.834 to 0.796 on average), while the elongation of the grains remains unchanged on average. HF etching also induces a significant reduction in mean grain size (between 17 and 29 µm) for three of the four samples. However, one sample remains largely unimpacted (C-E3888: reduction by <3 µm; Table S2) suggesting that the HF etching was (for an unknown reason) significantly less efficient in this case.

The reduction of the mean diameter measured by SIA indicates that the overall thickness removed by HF at a multi-grain scale ranges from ~1 to ~15 µm, with a mean etching depth (corresponding to half of the grain diameter reduction induced by HF etching) of ~8.5 µm. These values are systematically lower than the 20 ± 10 µm initially assumed^20^. They are also somewhat lower than other published values (e.g.^7^) for similar experimental conditions (HF 40% during 40 min), although based on a different approach (weight loss estimates). Although the maximum penetration range of alpha particles in silicate grains is usually considered to be ~20 µm^1^, published attenuation factors^37^ show that such an etching depth would not entirely remove the external alpha dose rate component (i.e. ~4% of the external alpha dose rate would remain). Observations in the present study show that HF etching has not successfully removed the full penetration depth of alpha particles from the quartz grains. In this instance, the alpha-irradiated outer layers have been dissolved by up to ~15 µm, but were significantly less for sample C-E3824, for which <2 µm had been removed from the grain surfaces. Consequently, the external alpha dose rate component should not be considered as null for the four Moulouya samples.

### Internal dose rate evaluations

Quartz grains are usually assumed to be virtually free of radioelements for evaluations of self-dosing effects in ESR dating. However, several studies have previously highlighted that there can be significant inter-sample variability in radioelement concentrations measured for purified quartz grains. For instance, very low values of 0.08 ± 0.02 and 0.18 ± 0.03 ppm for the U and Th concentrations, respectively, were reported for quartz grains from southern Netherlands^38^. Similarly, Australian quartz samples showed U and Th contents of 0.072 ± 0.005 and 0.238 ± 0.009 ppm, respectively^39^. Using the commonly employed a-value of 0.04 in OSL dating (e.g.^13,15^), the corresponding internal alpha dose rate was estimated to ~15 µGy/a^38^, which may be considered negligible for most ESR dating studies. However, with a higher alpha efficiency, the internal alpha dose rate would increase significantly, reaching ~25 µGy/a when using the experimentally-determined a-value of 0.07 and reaching ~75 µGy/a when assuming an a-value of 0.2. Finally, U, Th and K contents of 0.20–0.32 ppm, 0.09–0.14 ppm and 0.00–0.001%, respectively, were also reported for purified quartz samples from southeast Britain^40^. These quartz elemental concentrations would result in non-negligible internal dose rates of ~60 µGy/a for an experimental a-value of 0.07 and ~170 µGy/a for an assumed a-value of 0.2. These results show that internal alpha dose rate contributions should not be considered negligible, even though the measured radioelement concentrations are very low.

ICP-MS/OES analyses of quartz extracts (see details in Supplementary Information) from samples C-E3824 and C-E3888 reveal U and Th concentrations of 0.10–0.14 and 0.40–0.55 ppm, respectively (Table 2). These U and Th concentrations would result in an internal dose rate of 50–70 µGy/a when using our empirically determined a-value of 0.07 (Table 3). This range is very close to the internal dose rate of 50 ± 30 µGy/a previously assumed^20^ and would result in a small ESR age difference of <20 ka (Table 3). In contrast, samples C-E3886 and C-E3891 yielded U and Th concentrations ranging from 1.15–1.21 and 3.52–3.57 ppm, respectively, i.e. either similar to, or higher than, those measured in the raw sediment (Table 2). Such results are similar to “unexpected observations” made in previous studies (e.g.^41^). These authors show that some quartz samples contain significantly higher radioelement concentrations (up to several ppm of U and Th), sometimes of the same order of magnitude as in the raw sediment. According to^41^, this could be correlated to the presence of accessory minerals with high U-Th contents (e.g. opaque mineral, zircon, rutile; occurring either outside or as inclusions in the quartz grains), as suggested by the high concentration of other elements such as Zr or Fe. A similar pattern is observed in the Moulouya samples, as additional elemental analyses show significantly higher Al, K, Fe, Mg, Na and Ti concentrations for C-E3886 and C-E3891 (by a factor of >10) compared to C-E3824 and C-E3888 (Table 2). Microscope observations of the four Moulouya quartz samples did not reveal the presence of accessory minerals (i.e., other than quartz). Nevertheless, additional SEM analyses (see details in Supplementary Information) led to the identification of very small inclusions (<20 µm diameter) of rutile (Ti), mica (Mg, Al) and iron oxides (Fe) located at the surface of some quartz grains from samples C-E3886 and C-E3891. These inclusions may explain the higher elemental concentrations for these two samples (Table 2). However, the higher K contents can neither be explained by inclusions, nor by K-feldspar contamination, given the favourable IR depletion ratios^42^ measured in the associated luminescence dating study^32^.

**Table 2:** Element concentrations obtained from ICP-MS/OES analyses.

	Element concentrations									
	Element (unit)	U (ppm)	Th (ppm)	K (%)	Al (ppm)	Fe (ppm)	Mg (ppm)	Na (ppm)	Ti (ppm)	K (ppm)
	Detection level (ppm)	0.01	0.01	20	50	10	20	20	5	20
	Method	4 A/MS	4 A/MS	4A/MS	4ABSi/OE	4ABSi/OE	4ABSi/OE	4ABSi/OE	4ABSi/OE	4ABSi/OE
Sample ID	Material									
C-E3824	B	0.78	2.15	0.522	n.m.	n.m.	n.m.	n.m.	n.m.	n.m.
	Q	0.14	0.55	b.d.l.	2501	464	84	289	83	911
C-E3886	B	1.21	3.60	0.768	n.m.	n.m.	n.m.	n.m.	n.m.	n.m.
	Q	1.21	3.57	b.d.l.	58297	3329	1413	14629	1842	46341
C-E3888	B	0.80	3.29	0.791	n.m.	n.m.	n.m.	n.m.	n.m.	n.m.
	Q	0.10	0.40	b.d.l.	1359	282	51	129	62	300
C-E3891	B	0.75	2.60	0.669	n.m.	n.m.	n.m.	n.m.	n.m.	n.m.
	Q	1.15	3.52	b.d.l.	57485	3002	1090	12903	1707	50404

Key: B = bulk raw sediment, Q = purified quartz; n.m.: not measured; b.d.l.: below detection level. Method: 4 A/MS = Multi-acid digest/Inductively Coupled Plasma Mass Spectrometry analyses; 4 ABSi/OE = Multi-acid digest/Inductively Coupled Plasma Optical (Atomic) Emission Spectrometry analyses.

**Table 3:** Internal dose rate simulations based on ICP-MS/OES analyses and internal dose rate calculations using DRAC v1.2^50^. The large relative errors (>50%) on the internal dose rate values calculated for samples C-E3824 and C-E3888 are directly due to the low uranium concentrations measured by ICP-MS/OES in the quartz extracts (<0.2 ppm, i.e. close to the detection level; Table 2).

Sample ID	a = 0.07			a = 0.2 (Bartz *et al*.^20^)		
	Internal dose rate (µGy/a)	% of total dose rate	ESR age (ka)	Internal dose rate (µGy/a)	% of total dose rate	ESR age (ka)
C-E3824	66 ± 46	8.0	1250 ± 107	50 ± 30	6.1	1259 ± 96
C-E3886	496 ± 47	29.5	976 ± 96	50 ± 30	4.0	1312 ± 129
C-E3888	47 ± 62	4.1	1289 ± 115	50 ± 30	4.3	1269 ± 97
C-E3891	479 ± 46	34.7	856 ± 63	50 ± 30	5.2	1229 ± 98

If these high radioelement concentrations were to be considered in the dose rate evaluation of samples C-E3886 and C-E3891, they would yield a major internal dose rate of ~500 µGy/a with an a-value of 0.07. This internal dose rate component would represent 30–35% of the total dose rate for these two samples (Table 3) and would produce significantly younger ESR ages (by ~25–30%) in comparison with those previously calculated^20^. Although the resulting ages would still point to an Early Pleistocene chronology compatible with the independent age control produced by palaeomagnetic data, the stratigraphic interpretations of the ESR dating results become difficult to reconcile. In particular, sample C-E3891 has been collected from the uppermost unit, as in the adjacent section BOU (sample C-E3824)^20,32^, and an age difference of 0.3–0.4 Ma between the two ESR samples (C-E3891 and C-E3824) from these two sections, as well as with the independent single-grain TT-OSL ages obtained from the upper units of BOU^32^, would seem unrealistic. Consequently, the high concentration of U and Th obtained for C-E3886 and C-E3891 do not seem likely to be representative of the true internal dose rates. An assumed internal dose rate of 50 ± 30 µGy/a has therefore been maintained as the most reasonable estimate of the true internal dose rate for these samples; at least in the first instance until further analysis of the internal U and Th discrepancies can be undertaken.

Alternatively, it may be reasonable to consider the possibility of an inverse correlation between the concentration of radioelements in quartz and the alpha efficiency. This hypothesis has previously been proposed for tooth enamel and calcite (see^43^ and references therein). These earlier works have shown that some domains with high uranium concentrations may not necessarily correlate with high ESR/TL intensities, suggesting that highly ionizing alpha particles could induce more damage to the crystal lattice than the creation of paramagnetic centres. Similarly, fossil teeth with exceptionally high uranium concentrations in the enamel layer (>5 ppm) typically show systematic and significant age underestimations^44^, most likely due to an overestimation of the internal dose rate. Consequently, it might be envisaged that some of the domains showing high radioelement concentrations within a quartz grain may also locally display a lower alpha efficiency, thus lowering the overall internal dose rate. Similarly^45^, suggested that alpha efficiency might be affected by impurities arising along track damage in the mineral. However, further investigation is required to confirm this hypothesis, and ascertain its relevance for samples C-E3886 and C-E3891.

Finally, grain size and shape may also impact the internal dose rate. Based on SIA, the four samples mostly comprise non-spherical grains, which can be approximated as smoothed and slightly elongated ellipsoids. Unfortunately, it is currently difficult to quantify the impact of grain shape characteristics on our dose rate evaluations owing to the absence of suitable alpha attenuation factors in the literature, contrary to those available for the beta dose rate component^4,45^. The SIA grain size measurements can, however, be taken into account in the internal dose rate evaluations (assuming spherical grains): this leads to an increase of between 1% and 9% among the samples (6% on average) compared with the internal dose rate values displayed in Table 3. Similarly, when considering the measured etched depths (i.e., instead of the assumed 20 ± 10 µm), the resulting internal dose rates decrease by 8% on average. Consequently, these empirical amendments show compensating effects that eventually result in an overall slight decrease of the internal dose rate by about 3% on average compared to the values calculated earlier (Table 3).

### External alpha dose rate evaluations

Similar to the internal dose rate, one may expect the overall ellipsoidal shape of the grains to influence the external alpha dose rate^45^. However, this cannot be properly quantified at present owing to the absence of published alpha attenuation factors for non-spherical grains. The impact of grain size can be assessed for each sample by using the mean diameter and associated error experimentally measured by SIA (scenario #1, Table 4) in the dose rate evaluation. These new calculations result in external alpha dose rates contributing between 1.2 and 1.8% of the total dose rate, while the impact on the ESR age is relatively limited (<2% age difference with the ESR age results by^20^). When using the experimental alpha efficiency of 0.07 (Scenario #2, Table 4), the relative contribution of the external alpha dose rate drops below <1%, leading to ESR age estimates that are between 1% and 3% older than those published earlier.

**Table 4:** Grain size properties pre- and post-HF etching based on static image analysis and external alpha dose rate calculation using DRAC v1.2^50^.

Sample ID	Mean CE diameter pre-HF etching (µm)	Mean CE diameter post-HF etching (µm)	Etching depth (µm) ^(a)^	Scenario #1				Scenario #2				Scenario #3			
				External alpha dose rate (µGy/a)	% of total dose rate^(b)^	ESR age (ka)	ESR age ratio^(c)^	External alpha dose rate (µGy/a)	% of total dose rate^(c)^	ESR age (ka)	ESR age ratio^(c)^	External alpha dose rate (µGy/a)	% of total dose rate^(b)^	ESR age (ka)	ESR age ratio^(c)^
C-E3824	198.3 ± 1.7	181.1 ± 2.2	8.6 ± 0.1	10 ± 8	1.2	1281 ± 97	1.02	4 ± 3	0.5	1291 ± 97	1.03	9 ± 1	1.1	1277 ± 96	1.01
C-E3886	185.4 ± 9.1	156.5 ± 4.9	14.5 ± 0.8	18 ± 14	1.5	1330 ± 13	1.01	6 ± 5	0.5	1343 ± 135	1.02	7 ± 1	0.6	1341 ± 134	1.02
C-E3888	146.2 ± 1.1	143.5 ± 4.5	1.4 ± 0.04	21 ± 18	1.8	1270 ± 96	1.00	7 ± 6	0.6	128 ± 95	1.01	39 ± 5	3.3	1237 ± 91	0.98
C-E3891	185.4 ± 14.5	166.7 ± 4.0	9.4 ± 0.8	13 ± 10	1.4	1246 ± 98	1.01	4 ± 3	0.4	1257 ± 98	1.02	10 ± 2	1.1	1245 ± 97	1.01

Note that post-HF etching sieving has been performed using a 90 µm opening size sieve. ^(a)^calculated as the half of the difference in CE diameter, ^(b)^based on an internal dose rate of 50 ± 30 µGy/a, ^(c)^Compared to the original ESR ages published by Bartz *et al*.^20^.

Scenario #1: assumed alpha efficiency = 0.2; original grain size = measured mean CE diameter; assumed etching depths of 20 ± 10 µm.

Scenario #2: measured alpha efficiency = 0.07; original grain size = measured mean CE diameter; assumed etching depths of 20 ± 10 µm.

Scenario #3: measured alpha efficiency = 0.07; original grain size = measured mean CE diameter; measured etching depths.

Using the experimental a-value of 0.07 and the measured etching depths for each sample (Scenario #3, Table 4) would result in external alpha dose rate contributions of ~7 to ~39 µGy/a, which would represent between 0.6 and 3.3% of the total dose rate. Though relatively small, these alpha dose rate contributions are non-negligible. The impact on the final ESR ages for these four samples is <35 ka, which corresponds to a relative difference of <3% compared to the original age estimates^20^; (Table 4). The largest ESR age difference is observed for sample C-E3888, which exhibited unexpectedly ineffective etching effects, whereas the other samples are slightly impacted by <2%.

In summary, these results illustrate that variability of the HF etching rate may be observed between different samples, even within the same geographic area, which is consistent with previous observations^7^. This variability demonstrates the importance of systematically and experimentally determining the amount of material removed from the quartz grains by HF etching.

### Single grain considerations

**Figure 2:**
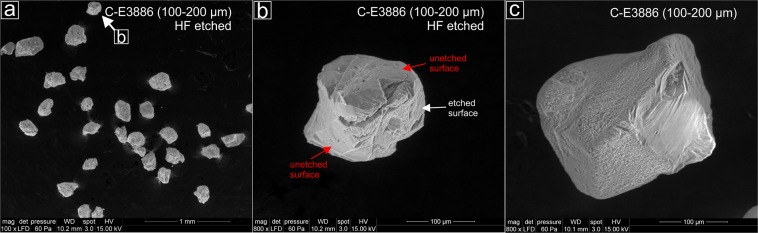
Scanning electron microscope (SEM) pictures of sample C-E3886 (100–200 µm). (**a**) A selection of different quartz grains post-HF etching; (**b**) Example of a quartz grain that does not show a uniform etch pattern at the grain surface. Red arrows mark surfaces that are unaffected by HF etching, while the white arrow shows typical marks of HF on the grain surface; (**c**) Example of a quartz grain pre-HF etching.

Additional SEM analyses of two quartz samples (see details in Supplementary Information) provides further insights into the effect of HF etching at a single grain level. Figure 2a,b provides an example of a 100–200 µm quartz grain from sample C-E3886 that was not uniformly etched, which is consistent with earlier observations^28,46^. For this sample, the HF-etched grains reveal surfaces that seems to have been largely unaffected by HF digestion and do not show typical etching patterns^47^; (Fig. 2a,b). It has been suggested that one potential reason for uneven HF etching might be different stages of mineral maturity^48^. This can significantly influence the solubility of HF on particle surfaces, meaning that the modal grain size of mineralogically less mature quartz particles is likely to be greatly reduced by HF exposure (30–50 µm) compared to that of more mature quartz grains (~10 µm)^48^. The two studied samples most likely originate from quartz-bearing Palaeozoic rocks in the Upper Moulouya catchment, located ~500 km upstream of the studied area (cf.^20,49^). Therefore, it can be assumed that the quartz samples deposited in the Lower Moulouya catchment are likely to be mineralogically mature^20,49^. This might explain why HF has affected the grain surfaces irregularly and only to a limited extent for some samples (Fig. 2; Table 4).

Despite these observations, it remains complicated to evaluate the extent to which such etching irregularities will influence the alpha dose rate component at a single grain level. As mentioned by^45^ (p.153), “alpha particles have a short range in relation to silt/sands sizes leading to a greater sensitivity to small-scale geometrical effects and consistent heterogeneities in the source distribution coupled with shape irregularities of the grain may lead to significant (and unknown) perturbations in the correction”.

## Conclusion

This study has presented the first experimental evaluation of the alpha efficiency value for ESR dating of quartz grains. Two samples from the Moulouya area (NE Morocco^20^) were analysed. They provided consistent results for the different irradiation doses and ESR signals considered. Based on these results, we recommend the use of an a-value of 0.07 ± 0.01 (1σ) for both the Al and Ti centres measured in coarse quartz grains. This value is significantly lower than those commonly assumed in ESR dating studies until now. We acknowledge that quartz ESR alpha efficiency may be sample dependent, possibly grain size dependent, or may locally vary within a grain due the presence of impurities or small-scale geometrical effects. However, it is difficult to evaluate this potential variability as there is presently no other experimental a-value available in the literature. This may be achieved in the future by performing similar experiments with quartz grains from different geographical origins, and using different grain sizes, as fine grains (typically 4–11 μm) fall within the range of the alpha particles contrary to coarse grains.

We experimentally assessed a series of parameters directly associated with the internal and external alpha dose rate components, such as radioelement concentrations, grain size and shape, and HF etching. In particular static image analysis appears to be a powerful tool to characterize the shape and size of the grains before and after HF etching. It also offers the possibility to quantify HF etching effects at a multi-grain level, probably more precisely than the usual approach based on weight loss. Our results show significant differences (and large variability) compared to the values that are typically assumed for these parameters, which directly impact the calculated internal and external alpha dose rates. However, because these two components typically have a limited weight in the total dose rate evaluation, the resulting impact of these experimental considerations on the ESR age estimates appear to be overall <5%.

## Supplementary information

Supplementary Information

## Data Availability

All data generated or analysed during this study are included in this published article (and its Supplementary Information file).
