# Publisher Correction: First experimental evaluation of the alpha efficiency in coarse-grained quartz for ESR dating purposes: implications for dose rate evaluation

**DOI:** 10.1038/s41598-020-62007-w

**Published:** 2020-03-18

**Authors:** Melanie Bartz, Lee J. Arnold, Nigel A. Spooner, Martina Demuro, Isidoro Campaña, Gilles Rixhon, Helmut Brückner, Mathieu Duval

**Affiliations:** 1 Institute of Geography, University of Cologne, Albertus-Magnus-Platz, 50923 Cologne, Germany; 2 School of Physical Sciences, Environment Institute, and Institute for Photonics and Advanced Sensing (IPAS), University of Adelaide, North Terrace Campus, Adelaide, SA 5005 Australia; 3 Defence Science and Technology Group, Third Avenue, Edinburgh, SA 5111 Australia; 4 Centro Nacional de Investigación sobre la Evolución Humana (CENIEH), Paseo Sierra de Atapuerca, 3, 09002 Burgos, Spain; 5 ENGEES/Laboratoire Image Ville Environnement (LIVE), UMR 7362 - CNRS (University of Strasbourg), Quai Koch 1, 67000 Strasbourg, France; 6 Australian Research Centre for Human Evolution (ARCHE), Environmental Futures Research Institute (EFRI), Griffith University, 170 Kessels Road, Nathan, QLD 4111 Australia

Correction to: *Scientific Reports* 10.1038/s41598-019-54688-9, published online 24 December 2019

This Article contains errors in Table 4.

Under column ESR age (ka) in scenario 1,

“1330±13”

should read:

“1330±134”

Under column ESR age (ka) in scenario 2

“128±95”

should read:

“1285±95”

Finally, there is an error in the heading “% of total dose rate^(c)^” where,

“% of total dose rate^(c)^”

should read:

“% of total dose rate^(b)^”

