# Supplementary material for: First experimental evaluation of the alpha efficiency in coarse-grained quartz for ESR dating purposes: implications for dose rate evaluation
Source: Sci Rep. 2019 Dec 24;9:19769. doi: 10.1038/s41598-019-54688-9 (PMC6930274; doi:10.1038/s41598-019-54688-9)
Supplement: Supplementary file 1 — Supplementary Information [file 41598_2019_54688_MOESM1_ESM.pdf]

SUPPLEMENTARY INFORMATION

First experimental evaluation of the alpha efficiency in coarse-grained quartz for ESR dating purposes: implications for dose rate evaluation

Melanie Bartz[1*], Lee J. Arnold[2], Nigel A. Spooner[2, 3], Martina Demuro[2], Isidoro Campaña[4], Gilles Rixhon[5], Helmut Brückner[1], Mathieu Duval[6, 4]

[1] Institute of Geography, University of Cologne, Albertus-Magnus-Platz, 50923 Cologne/Germany

[2] School of Physical Sciences, Environment Institute, and Institute for Photonics and Advanced Sensing (IPAS), University of Adelaide, North Terrace Campus, Adelaide, SA, 5005, Australia

[3] Defence Science and Technology Group, Third Avenue, Edinburgh, SA, 5111, Australia

[4] Centro Nacional de Investigación sobre la Evolución Humana (CENIEH), Paseo Sierra de Atapuerca, 3, 09002 Burgos/Spain

[5] ENGEES / Laboratoire Image Ville Environnement (LIVE), UMR 7362 - CNRS (University of Strasbourg), Quai Koch 1, 67000 Strasbourg/France

[6] Australian Research Centre for Human Evolution (ARCHE), Environmental Futures Research Institute (EFRI), Griffith University, 170 Kessels Road, Nathan, QLD 4111/Australia

*corresponding author: m.bartz@uni-koeln.de; +492214707719

Methods

Multi-element analyses

Multi-element analyses of both raw sediment and quartz extracts from four samples (C-E3824, C-E3886, C-E3888 and C-E3891) were performed by Genalysis Laboratory Services. U, Th and K concentrations were obtained by Inductively Coupled Plasma Mass Spectrometry analyses after a multi-acid digest preparation procedure (including Hydrofluoric, Nitric, Perchloric and Hydrochloric acids in Teflon Tubes). In comparison, the concentration of the other elements was derived from a multi-acid digest preparation procedure (including Hydrofluoric, Nitric, Perchloric and Hydrochloric acids in Teflon Beakers) followed by Inductively Coupled Plasma Optical (Atomic) Emission Spectrometry analyses. Numerical results are given in Table 2.

Dose rate evaluation

As mentioned above, the internal radioactivity of quartz grains was assessed through ICP-MS analyses of pure etched quartz from four samples. To ensure consistency with the earlier dating study by [1], the external dose rate values have been derived from the published radioelement concentrations obtained by high-resolution gamma-spectrometry (HRGS) analyses of the bulk sediment. The software DRAC v1.2 [2] was used for dose rate and age calculation with the conversion factors of [3], and the alpha and beta attenuation factors of [4] and [5] (specifically chosen for quartz), respectively. Water contents of 15±5% were used [1] and the cosmic dose rate contribution was assessed following the approach of [6].

Static Image analysis

Grain size analysis of quartz grains from four Moulouya samples (C-E3824, C-E3886, C-E3888 and C-E3891; unetched and etched quartz grains) were performed at CENIEH by static

image analyses (SIA) using a Malvern Instruments Morphologi G3 particle characterization system and Morphologi software 7.41, following the procedure outlined in [7]. With this technique, 3D particles are captured as high-resolution 2D images, from which various size and shape parameters can be derived:

- Circle Equivalent (CE) diameter is the diameter of a circle with the same area as the 2D image of the particle. Note that the etched grains were sieved with a 90 µm size opening sieve prior to the Static Image analyses. For a given sample, a CE diameter was thus obtained for each particle of the grain population and a mean value and associated error (1 standard deviation) were derived.

- Circularity: ratio of the perimeter of a circle with the same area as the particle divided by the perimeter of the actual particle image. This parameter allows quantifying how close the grain shape is to a perfect circle (a perfect circle has a circularity of 1).

- Aspect ratio: ratio of the width to the length of the particle. A perfect circle has a value of 1, whereas a rod would have a low value.

- Convexity: to evaluate the edge roughness of a particle. A smooth shape has a convexity of 1 while a very 'spiky' or irregular object has a convexity closer to 0.

Further details may be found in [8]. Numerical values are given in Table S2 and 4

Microscope analyses

High-resolution images of quartz grains were obtained at the CENIEH using a microscope FEI Quanta-600 in high vacuum, acting as a traditional scanning electron microscope (SEM). Two batches of hundreds of natural unetched and etched grains were analysed for samples C-E3886 and C-E3891 in order to visualize the impact of HF etching at a single grain scale. Furthermore, the presence of accessory minerals or inclusions was checked in the quartz

extracts of samples C-E3824, C-E3886, C-E3888 and C-E3891. Some examples of pictures are displayed in Figure 2.

Table S1: Normalised Al and Ti centre ESR intensities for the measured alpha- and gamma-irradiated aliquots of samples C-E3886 and C-E3891. The coefficient of variation (%) is also given. No baseline correction was performed. ESR intensities correspond to the mean value and associated 1 standard deviation derived from the repeated measurements.

Sample ID	Irradiation dose (Gy)	Number of ESR measurements	ESR intensity			
			Al centre	Ti centre (Option A)	Ti-H centre (Option C)	Ti centre (Option D)
C-E3886	2230±66 [1]	5	0.919±0.020 (2.2%)	0.864±0.035 (4.0%)	0.865±0.053 (6.1%)	0.880±0.036 (4.1%)
	200±5 [2]	5	1.081±0.020 (1.8%)	1.136±0.035 (3.0%)	1.135±0.053 (4.7%)	1.120±0.036 (3.2%)
	4460±132 [1]	2	0.899±0.049 (5.4%)	0.887±0.038 (4.3%)	0.976±0.018 (1.8%)	0.957±0.0004 (0.04%)
	395±9 [2]	2	1.101±0.049 (4.4%)	1.113±0.038 (3.4%)	1.024±0.018 (1.7%)	1.043±0.0004 (0.04%)
C-E3891	2230±66 [1]	4	0.914±0.011 (1.2%)	0.885±0.014 (1.6%)	0.954±0.059 (6.2%)	0.947±0.015 (1.5%)
	200±5 [2]	4	1.086±0.011 (1.0%)	1.115±0.014 (1.3%)	1.046±0.059 (5.6%)	1.053±0.015 (1.4%)
	4460±132 [1]	3*	0.917±0.015 (1.6%)	0.832±0.007 (0.8%)	0.817±0.027 (3.3%)	0.832±0.009 (1.1%)
	395±9 [2]	3*	1.083±0.015 (1.3%)	1.168±0.007 (0.6%)	1.183±0.027 (2.3%)	1.168±0.009 (0.8%)

[1] Alpha irradiation
[2] Gamma irradiation
* Only 2 measurements were considered for the Ti centre because of a very low S/N ratio due to the small amount of material measured, which precluded the extraction of meaningful values.

Table S2: Selected grain shape parameters obtained from static image analyses performed on samples C-E3824, C-E3886, C-E3888 and C-E3891 prior to and post HF etching. Mean values and associated errors (1σ-standard deviation) are also shown. The coefficient of variation (%) is given in brackets.

**Parameter**	**Sample ID**	**Pre HF**	**Post HF**	**Ratio**
**CE diameter**	C-E3824	198.3±1.7	181.1±2.2	
	C-E3886	185.4±9.1	156.5±4.9	
	C-E3888	146.2±1.1	143.5±4.5	
	C-E3891	185.4±14.5	166.7±4.0	
	Mean	178.8±23.6 (12.6)	161.9±15.9 (9.8%)	0.91
**Circularity**	C-E3824	0.843±0.004	0.799±0.009	
	C-E3886	0.834±0.006	0.781±0.009	
	C-E3888	0.833±0.004	0.806±0.001	
	C-E3891	0.827±0.024	0.798±0.006	
	Mean	0.834±0.007 (0.8%)	0.796±0.010 (1.3%)	0.95
**Aspect ratio**	C-E3824	0.762±0.007	0.759±0.003	
	C-E3886	0.764±0.006	0.762±0.003	
	C-E3888	0.769±0.007	0.784±0.001	
	C-E3891	0.759±0.004	0.769±0.004	
	Mean	0.764±0.004 (0.6%)	0.768±0.011 (1.5%)	1.01
**Convexity**	C-E3824	0.980±0.001	0.962±0.003	
	C-E3886	0.978±0.002	0.956±0.004	
	C-E3888	0.978±0.001	0.965±0.001	
	C-E3891	0.974±0.007	0.961±0.003	
	Mean	0.977±0.002 (0.2%)	0.961±0.004 (0.4%)	0.98

References

1. Bartz, M. *et al.* Successful combination of electron spin resonance, luminescence and palaeomagnetic dating methods allows reconstruction of the Pleistocene evolution of the lower Moulouya river (NE Morocco). *Quat. Sci. Rev.* **185**, 153–171 (2018).
2. Durcan, J. A., King, G. E. & Duller, G. A. T. DRAC: Dose Rate and Age Calculator for trapped charge dating. *Quat. Geochronol.* **28**, 54–61 (2015).
3. Guérin, G., Mericier, N. & Adamiec, G. Dose-rate conversion factors: update. *Anc. TL* **29**, 5–8 (2011).
4. Bell, W. T. Alpha dose attenuation in quartz grains for thermoluminescence dating. *Anc. TL* **12**, 4–8 (1980).
5. Guérin, G., Mercier, N., Nathan, R., Adamiec, G. & Lefrais, Y. On the use of the infinite matrix assumption and associated concepts: A critical review. *Radiat. Meas.* **47**, 778–785 (2012).
6. Prescott, J. R. & Hutton, J. T. Cosmic ray contributions to dose rates for luminescence and ESR dating: Large depths and long-term time variations. *Radiat. Meas.* **23**, 497–500 (1994).
7. Duval, M. *et al.* Assessing the uncertainty on particle size and shape: Implications for ESR and OSL dating of quartz and feldspar grains. *Radiat. Meas.* **81**, 116–122 (2015).
8. Morphologi G3 User Manual. *MAN0410, Issue 3.0.* (2010).
